# Supplementary material for: The 30-year evolution of oral cholera vaccines: A case study of a collaborative network alternative innovation model
Source: PLOS Glob Public Health. 2025 Jan 17;5(1):e0003599. doi: 10.1371/journal.pgph.0003599 (PMC11741658; doi:10.1371/journal.pgph.0003599)
Supplement: Attachment — Submitted filename: Response to Reviewers.docx [file pgph.0003599.s001.docx]

Manuscript PGPH-D-24-00562

Response to Reviewers

Dear Dr. Syed Shahid Abbas,

Thank you for providing us with the opportunity to submit a revised draft of the manuscript “The 30-year evolution of oral cholera vaccines: A case study of a collaborative network alternative innovation model” for publication in PLOS Global Public Health. We are grateful for the time, effort, and expertise that you and the reviewers shared, which we believe greatly helped to improve our manuscript. We have incorporated most of the suggestions made by the reviewers, visible in the track changes version, and have provided explanations in the following letter for any suggestions we did not take on board. Additionally, please see below, in green, a point-by-point response to the reviewers’ comments. All line numbers refer to the revised manuscript file with tracked changes.

We hope you will find that the manuscript is now suitable for publication in PLOS Global Public Health. Thank you again for your time and serious consideration, we look forward to your decision.

Very respectfully,

Kaitlin Large, on behalf of the authors

**Editor’s Comments to the Authors:**

Please consider both the reviewers' comments about clarifying the discussion of your findings. Is it also possible to provide the 'work-in-progress' references you have cited for reviewers to consider, or, alternatively, change them to publicly available documents?

**Author response:** Thank you for your comments! We have clarified the discussion of our findings by moving the “collaborative network innovation model” section and corresponding figure to the results section as suggested by Reviewer 1, which has thus simplified the discussion and conclusion section, per the reviewers’ recommendations. This section can now be found from lines 827 – 887 in the results section. The discussion and conclusion section now begins at line 888, and we believe it better synthesizes the findings of the manuscript.

In addition, we have removed the “work-in-progress” references that were previously cited, and instead added references to publicly available documents as suggested by you and the reviewers. Please find the new references listed on lines 122 – 123.

**Reviewers’ Comments to the Authors:**

**Reviewer 1**

This is a well written paper, and the research is strongly conducted. I really enjoyed reading it. It is an interesting story. It is great to document how vaccine development has been achieved by only a few individuals and how important collaborations are. I only have minor comments. I would say that my recommendation is between major and minor revision. It is not a lot that needs changing in my opinion.

**Author response:** Thank you very much for your comments and feedback, we really appreciate it!

1. Page 5, lines 108-109: It is not acceptable to reference papers in preparation as these cannot be looked up by the reader. There are indeed already published papers on drug development within neglected disease. Please reference some of these instead as that would be useful for the reader to know about.

**Author response:** Thank you for pointing this out. The reviewer is correct, and we have removed the “work-in-progress” references that were previously cited, and instead replaced them with relevant publicly available documents. Please find the new references listed on lines 122 – 123.

2. Please check through that all abbreviations are spelled out the first time you mention them. Lines 153 and 154: I don't think we define IVI and ROK.

**Author response:** Thank you for pointing this out. The abbreviations for IVI and ROK originally on lines 153 and 154 have been changed to spell out “International Vaccine Institute (IVI)” and “Republic of Korea (ROK)” on lines 172 and 173. We confirm that the remainder of abbreviations throughout the paper are now spelled out upon first mention.

3. Lines 151-154. Please state how many interviews were undertaken with each of the mentioned methods. How many on video, how many in person?

**Author response:** As suggested by the reviewer, we have specified that eight interviews were conducted via video conference platform, nine interviews were conducted in-person, and one interview was conducted via email. These numbers have been added to lines 172 – 175.

4. Line 174: The analytical framework is mentioned as 'developed for the broader research project'. Is this the same as CAPIS mentioned earlier? It is necessary to describe the framework in this paper as this is what you use for data analysis. Please add this.

**Author response:** Yes, this analytical framework is the same as the CAPIS mentioned earlier. We have now added the “CAPIS” descriptor to line 196. The CAPIS conceptual framework developed for the research project is currently under peer review for publication as a separate academic journal article. We suggest updating a reference to this paper at a later time.

5. Table 1: In the column on 'Developers', please add the country the developers are based in. This is so it is easier for the reader to align the figure and the table - and easier to remember who is who.

**Author response:** As suggested by the reviewer, we have added the country of each developer to the “developer” column within Table 1, which can be found on lines 250 – 258.

6. Line 258: SIDA is the Swedish Government Development Agency and not something you can be a 'member' of. Was he a staff at SIDA? Or does SIDA have an advisory Board?

**Author response:** Thank you for pointing this out. The reviewer is correct, and SIDA had an advisory board which Prof. Holmgren was a member of at the time. We have made this clarification to the manuscript, which can be found on line 290.

7. Line 336: Gavi was established in year 2000, so it is confusing that it is mentioned already here in a section about 1997. The stockpile of course came much later.

**Author response:** As suggested by the reviewer, we have removed the mention of Gavi from line 382. While it was in reference to the fact that Dukoral would later become eligible for Gavi procurement, we agree that it is confusing to the reader.

8. Line 561: Unclear what 'drug product' is being referred to since the topic is vaccines

**Author response:** Thank you for pointing this out. We have changed “drug product” to “vaccine” on line 625.

9. It is stated in line 511 that IVI received grant from BMGF to organize two technology transfers. You only describe the one to EuBiologics. Please clarify if they are working on another one as part of this agreement.

**Author response:** As suggested by the reviewer, we have clarified that as part of the BMGF grant to IVI, the first technology transfer was conducted with Shantha for the production of Shanchol, while the second technology transfer was conducted with EuBiologics for the development of Euvichol, which can be found on lines 573-574.

10. Lines 567-569: Please explain why a phase III trial is necessary for EuBiologics. Is a phase III trial necessary when it is tech transfer of a vaccine where efficacy has already been shown? You mention in line 569 that the trial showed similar immunogenicity to Shanchol. I don't think that a phase III trial is needed to show immunogenicity. is that not only a phase II?

**Author response:** Thank you for your question. A phase III trial was required to compare the safety and efficacy of the new EuBiologics’ produced treatment (Euvichol) against the current standard treatment (Shanchol). Although the technology for the vaccine production is the same, a new producer needs to demonstrate that their vaccine has the same and/or a better safety and efficacy profile as the existing treatment. In addition, Phase III trials are also needed to show immunogenicity.

11. Line 640: 'Currently' is repeated twice

**Author response:** Thank you for pointing this out. The repeated word “currently” has been removed and corrected on lines 709-710.

12. You cannot include new findings in the Discussion and Conclusion section. It is not recommended to include a figure in this section. The figure and the accompanying text should be moved to the results section. This will also make the discussion an appropriate length. It is too long as it is. I recommend that the discussion starts from line 814.

**Author response:** We agree with your assessment and think this is a great suggestion. We have moved the “collaborative network innovation model” section and corresponding figure to the results section, which can be found on lines 827 – 887. The discussion and conclusion section now starts from line 888, per your recommendation.

13. Lines 837-842: Please add references for Moonshot initiative and READDI. It cannot be assumed that the reader knows about this.

**Author response:** As suggested by the reviewer, we have added references for the Moonshot initiative and READDI, which can be found on lines 1049 – 1050.

**Reviewer 2**

Thank you for the opportunity to review this interesting article "The 30-year evolution of oral cholera vaccines: A case study of a collaborative network alternative innovation model.” The manuscript tells the story of the long road to our current oral cholera vaccines and provides a great perspective on how other vaccines for diseases of the poor could proceed. This story will be a nice contribution to the literature, and I only have a couple ‘major’ comments along with several minor suggestions/questions.

**Author response:** Thank you very much for your assessment and recommendations, we really appreciate it!

**Major comments:**

1. The participatory network maps are really wonderful, but I think it would be helpful for the authors to provide more guidance on how to interpret them (e.g., do edge lengths matter? coloring of edges? size of nodes? placement of nodes?). Can we assume that these maps capture all the direct transfers of funding between donors and recipient? For example, in Figure 7 BMGF only gave money directly to WHO and IVI? Wellcome to IVI and U. of Gothenburg? I think the authors should double check each of these (some small comments below) as think there may be some omissions/mistakes.

**Author response:** Thank you for your feedback, and we think this is a very important recommendation! We have added additional guidance on how to interpret the participatory network maps to lines 314 – 319, specifically addressing the examples you provided, and explaining that they capture the direct transfers of funding between donors and recipients, as well as knowledge sharing and manufacturing flows. Furthermore, we have reviewed and addressed your additional comments below, thank you for pointing these out.

2. The actual use of OCV in emergencies and outbreaks played an important role in the story yet I feel like this was largely ignored. The first use of the Dukoral in a humanitarian setting was with Dukoral in 1999 (<https://www.ncbi.nlm.nih.gov/pmc/articles/PMC2557739/pdf/10593032.pdf>) which showed feasibility. It is important to note that those involved in this original deployment went on to lead the WHO Cholera Team at WHO (Dominique Legros) who helped to create a policy environment ripe for development and which opened the door to the ICG processes. Around the same time as Shanchol was being PQ’d MSF (Doctors without Borders) was the first to use this vaccine reactively (<https://www.nejm.org/doi/full/10.1056/NEJMoa1312680>) using their own funds. MSF has a seat on the ICG, and this use paved the way to greater acceptance of the potential role of reactive vaccine use. The first deployment of the stockpile was in South Sudan (<https://journals.plos.org/plosmedicine/article?id=10.1371/journal.pmed.1001901>) again led by MSF. I suspect that this part of the story is missing partly because of the snowball sampling used (which can lead you to stay within a tight network) and strongly urge you to consider talking to others involved in this side of the story.

**Author response:** Thank you for this suggestion. It would have been very interesting to further expand upon the actual use of OCVs in emergencies and outbreaks. Unfortunately, given the time constraints and resources available for the existing research project, we consider interviewing stakeholders beyond those who were directly involved in the R&D side of OCVs to fall beyond the scope of our case study. Nevertheless, we are grateful to you for providing this additional information on the role of key individuals and MSF in expanding the evidence base for changing WHO guidelines on the use of OCVs for cholera outbreaks, and we have incorporated information from these three references into the manuscript. This information can be found on lines 338 – 345, 533 – 537, and 544 – 548.

**Minor points:**

1. The introduction states that incidence of cholera outbreaks has been rapidly increasing and this is largely due to population displacement caused by conflicts and climate change. While this is a convenient narrative (and something that WHO has more or less said), this is a scientific manuscript and I urge you to find evidence to support this statement or remove/tone it down. Both the ‘increase in outbreaks’ (not just changes in surveillance and willingness to report) and the link with climate change are debatable.

**Author response:** As suggested by the reviewer, we have removed this statement in both the abstract and introduction, which can be found on lines 25 and 51.

2. L72 - I recommend you refer to the new surveillance guidelines for definition of an outbreak (<https://www.gtfcc.org/wp-content/uploads/2023/02/gtfcc-public-health-surveillance-for-cholera-interim-guidance.pdf#page=11.07>)

**Author response:** Thank you for providing this link. As suggested by the reviewer, we have updated the definition of an outbreak according to the new surveillance guidelines provided. This change can be found on line 71.

3. L75-78- The situation is a little more complex than suggested in the text. The ICG only controls the emergency stockpile of vaccines for outbreaks and other emergency situations. The rest of the OCV supply is purchased and controlled by Gavi (for use in multi-year vaccination plans that are typically considered preventive). This seems to be mentioned late in the manuscript (e.g., L708) but that is a bit late.

**Author response:** Thank you for pointing this out. As suggested by the reviewer, we have added this more nuanced description to lines 82 – 87.

4. L87 - To be clear there has never really been a time when we didn’t have a shortage of OCV in the world since it has been PQ’d.

**Author response:** Thank you for pointing this out. We have added further description to clarify that the shortage of OCVs has been a persistent problem since WHO PQ, to lines 94-95.

5. L93-95 - Probably want to update now that Euvichol-S is PQ’d and being produced

**Author response:** Thank you for your suggestion. We have updated the text to include information about Euvichol-S to lines 109 – 111.

6. L108-109 - I don’t think you can typically cite manuscripts in prep since no one can actually see/read these.

**Author response:** Thank you for pointing this out. The reviewer is correct, and we have removed the “work-in-progress” references that were previously cited, and instead replaced them with relevant publicly available documents. Please find the new references listed on lines 122 – 123.

7. L129-130 - Can you please add this as supplemental materials rather than asking people to request from author?

**Author response:** The CAPIS conceptual framework paper developed for the research project is currently under peer review for publication as a separate academic journal article. We suggest updating a reference to this paper in the manuscript at a later time.

8. L234 / 236 - Check references. Not sure what I_6 refers to.

**Author response:** Thank you for pointing this out. I_6 refers to “Interviewee 6”. We have tried to clarify the use of these abbreviations in the Methods section, line 172.

9. Figure 2: In lines 276:279 USAID is mentioned as a major donor, yet they are not in Figure 2. Is this intentional?

**Author response:** Yes, the exclusion of USAID in Figure 2 was intentional, as they were strictly a funder of icddr,b, and thus the inclusion of icddr,b in the figure attempts to by proxy represent their various funders as well, while maintaining the readability of the figure. Please let us know if we should redact the mention of USAID to help facilitate ease of reading.

10. L291 - “autochthonous cases” meaning what exactly?

**Author response:** Thank you for highlighting this and asking for clarification. We agree that the term used in this context is confusing, and we have removed the term from line 327, and instead specified that Dukoral used a dual market strategy to earn revenue on the traveler’s market, which then aided Dukoral’s ability to be made available on the public market.

11. L317 - mention the strain name.

**Author response:** Line 363 has been updated to include the strain name “Vibrio cholerae 0139”.

12. L355 - As far as I recall, the Mozambique study only measured outcomes in the first 4-5 months after vaccination. Also double check Zanzibar duration. Not sure it was longer than 1.5 years.

**Author response:** Thank you for pointing this out. The reviewer is correct, and we have updated these timeframes for the Mozambique and Zanzibar studies and cited the appropriate references, which can be found on lines 402 – 405.

13. Figure 5: what does the red outline of SIDA represent here? Also for Figure 5, can you confirm that no funds went from Gates to Shanta directly?

**Author response:** Thank you for pointing this out. The red outline of SIDA is a mistake, and we have removed it from the updated version of Figure 5. In addition, we confirm that no funds went from Gates to Shantha directly, but rather, Gates funded IVI to engage in tech transfer with two manufacturers, and Shantha was the first of the two manufacturers (in addition to later, EuBiologics) that they engaged in tech transfer with.

14. Table 3: It seems important to point out that the Global Health Investment Fund is something started by Gates. This now makes the foundation donors to this larger project and investors.

**Author response:** Thank you for pointing this out. We have added this distinction to the table, which can be found on line 610.

15. Figure 6: I don’t see any direct links between IVI and EuBiologics here. Is this intentional? Also not clear to me what the colored edges between IVI —> U Gothenburg —> EuBiologics means (same for IVI —> Jan H. —> Shantha)?

**Author response:** Thank you for pointing this out. It was an accident due to the placement of the nodes in the software program, which disguised the direct link between IVI and EuBiologics. We have added the direct link between IVI and EuBiologics to the updated version of Figure 6.

In addition, the ombre edges between these different entities are not intentional, but rather automatically created by the software program. We have clarified in the manuscript that the ombre edges do not hold additional meaning, which can be found in lines 314 – 319.

16. Line 601: Who is this the cost for? The stockpile or anyone?

**Author response:** Thank you for asking for this clarification. This is the price for the stockpile. We have clarified this in the text, which can be found on lines 667 – 668.

17. Since submitting this manuscript, Euvichol-S has been PQ’d and is going to be shipping soon. I suggest you rework the way Euvichol-S is discussed in light of this news.

**Author response:** Thank you for pointing this out. Throughout the manuscript, we have revised how Euvichol-S is discussed.

18. Table 1:Might be useful to add a column indicating availability and/or stage of development. This table includes vaccines no longer produced and those not yet in clinical studies. In the product characteristics there are data on efficacy for each. Are these from package inserts or generally from the 2021 review paper in Vaccine? I would suggest citing the original sources for the data for each instead. Do note that this is a hard comparison since OCV protection wanes with time.

**Author response:** We think this is a great suggestion. We have added a column to Table 1 titled “Availability/stage of development” and included the relevant information for each vaccine. We also agree with the reviewer’s comment that the comparison of efficacy data is difficult to make for each vaccine, as protection wanes with time. Therefore, we have also removed the efficacy data. The changes made can be found on lines 251 – 259.
