# Supplementary material for: The 30-year evolution of oral cholera vaccines: A case study of a collaborative network alternative innovation model
Source: PLOS Glob Public Health. 2025 Jan 17;5(1):e0003599. doi: 10.1371/journal.pgph.0003599 (PMC11741658; doi:10.1371/journal.pgph.0003599)
Supplement: Attachment — Submitted filename: Response_to_Reviewers_auresp_2.docx [file pgph.0003599.s002.docx]

Manuscript PGPH-D-24-00562

Response to Reviewers

Dear Dr. Syed Shahid Abbas,

Thank you for providing us with the opportunity to submit a revised draft of the manuscript “The 30-year evolution of oral cholera vaccines: A case study of a collaborative network alternative innovation model” for publication in PLOS Global Public Health. We are grateful for the time, effort, and expertise that you and the reviewers shared, which we believe greatly helped to improve our manuscript. We have incorporated the final clarification from Reviewer #2, which is visible in the track changes version of the manuscript. Additionally, please see below, in green, a point-by-point response to the reviewers’ comments. The line numbers refer to the revised manuscript file with tracked changes.

We hope you will find that the manuscript is now suitable for publication in PLOS Global Public Health. Thank you again for your time and serious consideration, we look forward to your decision.

Very respectfully,

Kaitlin Large, on behalf of the authors

**Editor’s Comments to the Authors:**

I agree with both the reviewers' comments about the authors' efforts to incorporate the reviewer comments. I would be happy to recommend this manuscript for publication after you address the clarification from Reviewer 2 (about Hillchol vaccine being licensed in India).

**Author response:** Thank you very much for your comments, feedback, and support in recommending this manuscript for publication! We are grateful for your time and consideration!

**Reviewers’ Comments to the Authors:**

**Reviewer 1**

All comments have been addressed well. I congratulate the authors for an excellent paper.

**Author response:** Thank you very much for your comments and feedback which greatly helped to improve the manuscript, and for your kind words, we really appreciate it!

**Reviewer 2**

The authors did a nice job responding to the reviewer comments and I believe the manuscript is in good form to be published. Only one small thing to update before publication is that the Hillchol vaccine is now licensed in India whereas the table in the manuscript says it has gone through a Phase II trial.

**Author response:** Thank you very much for your kind words and your support for publication! Also, thank you for providing this clarification. We have updated the manuscript to include that the Hillchol vaccine has been licensed in India, which can be found on line 233 and lines 712-713. Thank you for all of your time, comments, and feedback which greatly helped to improve the manuscript!
